# Estimation of salt intake from spot urine samples in patients with chronic kidney disease

**DOI:** 10.1186/1471-2369-13-36

**Published:** 2012-06-08

**Authors:** Makoto Ogura, Ai Kimura, Koki Takane, Masatsugu Nakao, Akihiko Hamaguchi, Hiroyuki Terawaki, Tatsuo Hosoya

**Affiliations:** 1 Division of Kidney and Hypertension, Department of Internal Medicine, The Jikei University School of Medicine, 3-19-18, Nishi-shinbashi, Minato-ku, Tokyo, 105-8471, Japan

**Keywords:** Chronic kidney disease, Salt intake, Urinary sodium excretion

## Abstract

**Background:**

High salt intake in patients with chronic kidney disease (CKD) may cause high blood pressure and increased albuminuria. Although, the estimation of salt intake is essential, there are no easy methods to estimate real salt intake.

**Methods:**

Salt intake was assessed by determining urinary sodium excretion from the collected urine samples. Estimation of salt intake by spot urine was calculated by Tanaka’s formula. The correlation between estimated and measured sodium excretion was evaluated by Pearson´s correlation coefficients. Performance of equation was estimated by median bias, interquartile range (IQR), proportion of estimates within 30% deviation of measured sodium excretion (P_30_) and root mean square error (RMSE).The sensitivity and specificity of estimated against measured sodium excretion were separately assessed by receiver-operating characteristic (ROC) curves.

**Results:**

A total of 334 urine samples from 96 patients were examined. Mean age was 58 ± 16 years, and estimated glomerular filtration rate (eGFR) was 53 ± 27 mL/min. Among these patients, 35 had CKD stage 1 or 2, 39 had stage 3, and 22 had stage 4 or 5. Estimated sodium excretion significantly correlated with measured sodium excretion (R = 0.52, P < 0.01). There was apparent correlation in patients with eGFR <30 mL/min (R = 0.60, P < 0.01). Moreover, IQR was lower and P30 was higher in patients with eGFR < 30 mL/min. Estimated sodium excretion had high accuracy to predict measured sodium excretion, especially when the cut-off point was >170 mEq/day (AUC 0.835).

**Conclusions:**

The present study demonstrated that spot urine can be used to estimate sodium excretion, especially in patients with low eGFR.

## Background

Chronic kidney disease (CKD) is characterized by hypertension, which is thought to be predominately salt sensitive [1]. High salt intake in CKD patients may cause high blood pressure, increased albuminuria and increased filtration fraction [2-7]. Salt restriction can result in diminished albuminuria and preservation of renal function [8-10]. The INTERSALT study [11] estimated salt intake by 24 h urine collection at 52 centers in 32 countries, and showed that the mean daily salt intake was 9.2 g. However, the average estimated salt intake by 24 h urine collection obtained from 3 Japanese centers was 11.0 g, suggesting that the Japanese have a relatively higher than average salt intake. The major methods in current use for assessment salt intake are dietary recall and 24 h urine sodium excretion measurement. Dietary recall is often inaccurate, and many patients are truly unaware of the amount of sodium they consume. Measurement of 24 h urine sodium excretion is considered to be reliable, and has been used in many clinical and epidemiological studies, including the INTERSALT study. However, it is relatively difficult to perform because of the necessity of 24 h urine sampling, and inadequate urine pooling leads to under- or over-estimation of salt intake. The use of a spot urine method, or a brief timed collection, for assessing sodium excretion has previously been examined [12-14]. Tanaka et al. [12] developed a simple method to estimate 24 h urinary sodium excretion from spot urine specimens collected at any time, using 591 Japanese data items from the INTERSALT study. Estimated sodium excretion was significantly correlated with measured sodium excretion using 24 h urine collection among these populations. There have been few reports about estimated salt intake in patients with a decline in renal function. The purpose of the present study was to evaluate whether the estimation of salt intake from spot urine could predict real salt intake in patients with CKD.

## Methods

A total of 100 CKD subjects were recruited from the outpatient department of The Jikei University Kashiwa Hospital between October 2009 and May 2010. All patients were requested to collect urine for 24 h. Salt intake was assessed by determining urinary sodium excretion from the collected urine samples. Furthermore, spot urine samples were examined on the same day that patients brought their 24 h urine samples to the hospital. These measurements were performed at least twice during the study period. We also measured urinary creatinine (Cr) excretion using 24 h urine samples. Data were excluded when urinary Cr excretion <1000, >2500 mg/day for male and <600, >1500 mg/day for female considering inadequate urine collection [15]. Four patients were excluded because of inadequate collection of urine. Estimation of sodium excretion by spot urine was calculated by Tanaka’s method, using the following formula [12].

(1) $$\begin{array}{l} 24\;\text{h Na excretion}\;\left(\text{mEq}/d\right)=21.98\times {\left\{\left[\text{Na S}/(\text{Cr S}\times 10,)\right]\times \text{Pr}.\text{UCr24}\;\right\}}^{0.392}\\ \;\text{Na S}:\text{Na concentration in spot urine}\;\left(\text{mEq}/L\right)\\ \;\text{Cr S}:\text{Cr concentration in spot urine}\;\left(\text{mg}/\text{dL}\right)\\ \;\text{Pr}.\text{UCr}24:\text{predicted value of}\;24\;\text{h urinary Cr excretion}\;\left(\text{mg}/d\right)\\ =-2.04\times \text{Age}\left(\text{years}\right)+14.89\times \text{Bodyweight}\left(\text{kg}\right)\\ +16.14\times \text{Height}\left(\text{cm}\right)-2244.45 \end{array}$$

Briefly, 24 h sodium excretion (24HUNaV) (mEq/day) was estimated by the formula as follows; 21.98 × XNa^0.392^ (XNa = Na concentration in the spot urine / creatinine concentration in the spot urine × predicted value of 24 h urinary Cr excretion (Pr.UCr24)). This formula including constant was obtained using 591 Japanese data items from the INTERSALT study.

Estimated values of urinary sodium excretion calculated using spot urine were compared to sodium excretion assessed by 24 h urine samples. The correlation between estimated and measured sodium excretion was evaluated by univariate analysis. The values of salt intake were calculated from urinary sodium excretion by standard method (1 mEq of sodium = 58.5 mg of NaCl). Correlations between estimated and measured sodium excretion were evaluated by Pearson´s correlation coefficients. The median difference between measured and estimated sodium excretion was used to calculate the bias. Precision of the estimates was expressed by interquartile range (IQR). Accuracy was measured as the proportion of estimates within 30% deviation of measured sodium excretion (P_30_). Accuracy was also expressed in terms of the root mean square error (RMSE). The Bland-Altman Plot was used to estimate the bias and limits of agreement between measurements by the two methods. To analyze the sensitivity and specificity of estimated sodium excretion in relationship to measured sodium excretion, receiver-operating characteristic (ROC) curves were generated, including area under the curve (AUC) and their 95% confidence intervals (CI). The sensitivity and specificity of estimated against measured sodium excretion were separately assessed by two cut-off points (>170 and >100 mEq/day, respectively).

The protocol was in conformity with institutional ethical guidelines (The Ethical Committee of The JIkei university school of medicine) and informed consent was obtained from each participant. Data were analyzed by using SPSS 17.0 (SPSS Inc, Chicago, IL), Bland-Altman with MedCalc software (version 12.1.4.0; Mariakerke Belgium). The P values reported are 2-sided and were taken to be statistically significant at 0.05.

## Results

Table 1 shows the characteristics of patients. Mean age was 58 ± 16 years and 49% of the patients were men. Mean serum Cr was 1.4 ± 1.0 mg/dL and estimated glomerular filtration rate (eGFR) was 53 ± 27 mL/min. Among these patients, 35 had stage 1 or 2 CKD (36.5%), 39 had stage 3 CKD (40.6%), and 22 had stage 5 CDK (22.9%). Mean urinary protein excretion was 1.2 ± 1.4 g/day.

**Table 1:** Clinical characteristics

**Characteristics**	**n = 96**
Age (years)	58 ± 16
Gender (male, %)	47 (49)
Cr (mg/dL)	1.4 ± 1.0
eGFR (mL/min)	53 ± 27
CKD stage (n, %)	
stage 1,2	35 (36.5)
stage 3	39 (40.6)
stage 4,5	22 (22.9)
UPR (g/d)	1.2 ± 1.4
Urine volume (mL/d)	1889 ± 547

eGFR: estimated glomerular filtration rate, UPR: urinary protein excretion.

**Figure 1:**
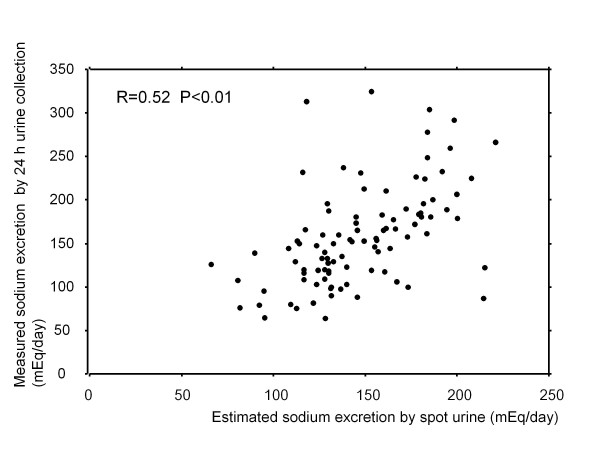
**Correlation between estimated sodium excretion by spot urine and measured sodium excretion assessed by 24 h urinary samples.** Estimated sodium excretion significantly correlated with measured sodium excretion (R = 0.52, P < 0.01)

**Figure 2:**
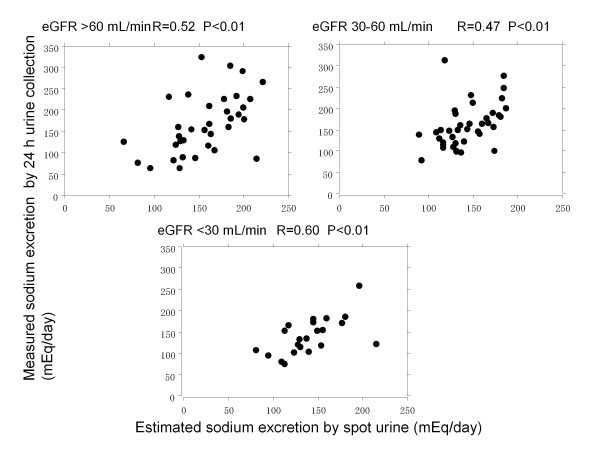
**Correlation between estimated sodium excretion and measured sodium excretion categorized by eGFR.** Significant correlations were recognized between estimated and measured sodium excretion in patients with eGFR >60 and 30–60 mL/min (R = 0.52, P < 0.01 and R = 0.47, P < 0.01, respectively). There was apparent correlation in patients with eGFR <30 mL/min (R = 0.60, P < 0.01)

A total of 334 samples from 96 patients were examined. Figure 1 shows the correlation between estimated sodium excretion by spot urine and measured sodium excretion assessed by 24 h urine sampling. Estimated sodium excretion significantly correlated with measured sodium excretion (R = 0.52, P < 0.01). Figure 2 demonstrates the correlation between estimated and measured sodium excretion categorized by eGFR. Significant correlations were recognized between estimated and measured sodium excretion in patients with eGFR >60 and 30-60 mL/min (R = 0.52, P < 0.01 and R = 0.47, P < 0.01, respectively). There was apparent correlation in patients with eGFR <30 mL/min (R = 0.60, P < 0.01).

**Figure 3:**
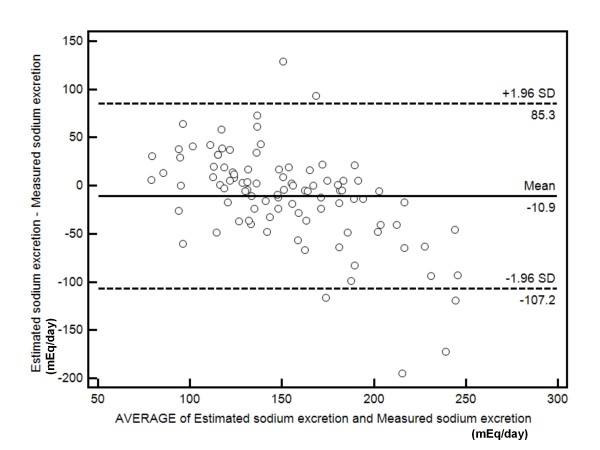
**Bland-Altman analysis of the differences between estimated and measured sodium excretion compared to the average sodium excretion by the two methods.** The mean difference was -10.9 (central line). A total of 94% (90 of 96) of the values lie within 1.96 SDs of the mean (outer lines)

Figure 3 shows Bland-Altman analysis of the differences between estimated and measured sodium excretion compared to the average sodium excretion by the two methods. The mean difference was -10.9 (central line). A total of 94% (90 of 96) of the values lie within 1.96 SDs of the mean (outer lines).

**Figure 4:**
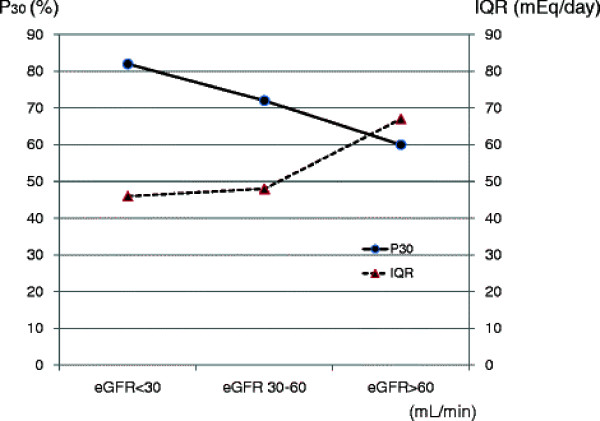
**Accuracy and precision of estimates expressed as P30 and IQR categorized by eGFR.** Highest accuracy within 30% and lowest precision were found for samples with an eGFR <30 mL/min. P30: percentage of estimated sodium intake within 30% of measured sodium intake, IQR: interquartile range

Table 2 demonstrated performance of urinary sodium estimating equation in patients with CKD. Median bias was 4.5 and percentage bias was 2.7%. IQR, an indicator of precision, was 53 mEq/ day. RMSE and P30, an indicator of accuracy, were 50 mEq/ day and 70%, respectively in overall patients. For each of the eGFR equations, the dataset was split into 3 groups: eGFR <30, 30–60, and >60 mL/min. Median bias and IQR were lower in patients with eGFR < 30 mL/min. Moreover, RMSE was lower and P30 was higher in patients at lower levels of eGFR (Table 2, Figure 4).

**Table 2:** Performance of urinary sodium estimating equation

**eGFR**	**n**	**Median bias**	**Median percentage bias**	**IQR**	**RMSE**	**P30**
**mL/min**		**(95% CI)**	**%**	**mEq/day**	**mEq/day**	**%**
eGFR <30	22	0.0 (-14.2-24.1)	0.0	46	34	82
eGFR 30-60	39	9.0 (-1.0-19.4)	5.9	48	48	72
eGFR >60	35	-2.0 (-5.8-16.3)	-1.3	67	60	60
Overall	96	4.5 (-2.1-12.2)	2.7	53	50	70

eGFR: estimated glomerular filtration rate, IQR: interquartile range, RMSE: root mean square error, P30: percentage of estimated sodium intake within 30% of measured sodium intake.

**Figure 5:**
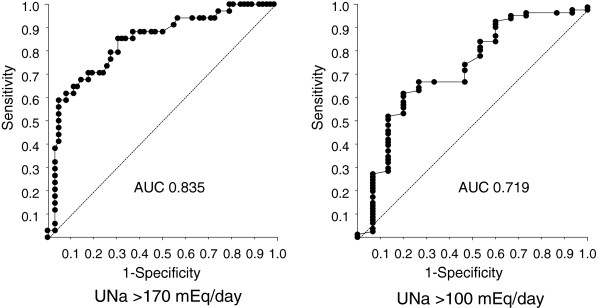
**ROC curves, including area under the curve (AUC).** The sensitivity and specificity of estimated sodium excretion against measured sodium excretion was separately assessed by two cut-off points (>170 and >100 mEq/day, respectively). Estimated sodium excretion had high accuracy to predict measured sodium excretion using AUC, especially when the cut-off point was >170 mEq/day (AUC 0.835). UNa: urinary sodium excretion

Finally, AUC values of estimated sodium excretion for predicting measured sodium excretion were examined by ROC curves. As shown in Figure 5, the AUC values were 0.835 and 0.719, respectively, when the cut-off point was >170 mEq/day and >100 mEq/day. Estimated sodium excretion had high accuracy to predict measured sodium excretion, especially when the cut-off point was >170 mEq/day.

## Discussions

The present study indicated that estimated sodium excretion from spot urine collected from CKD patients had adequate value for prediction of measured sodium excretion from 24 h urine collection. The use of a spot urine method or another brief timed collection for assessment of sodium excretion has previously been examined [12-14]. Kamata et al. [13] predicted 24 h sodium excretion using overnight urine by pipe-sampling method. Pr.UCr24 was estimated from lean body mass. Sodium excretion in nighttime urine has been reported to be relatively close agreement with the value determined in 24 h urine collection. Kawasaki et al. [14] estimated urinary sodium excretion, using Na/Cr concentrations in the second urine sample collected after waking. An estimate of Pr.UCr24 can be calculated from a formula height, body weight and age. However, its clinical use may be limited because second urine sampling must be performed after waking and before breakfast. Estimation of salt intake using a spot urine sample collected at any time would be much easier to perform. Unfortunately, the spot urine Na/Cr ratio has inherent limitations in estimating 24 h sodium excretion because sodium excretion varies considerably from hour to hour. And, the accuracy of predicting 24 h sodium excretion is altered by interindividual differences in 24 h creatinine excretion. Tanaka et al. [12] reported a simple method that can be used to estimate 24 h urinary sodium excretion from spot urine specimens that are collected at any time using 591 Japanese data items from the INTERSALT study. Estimated sodium excretion calculated by the formulas shown in Table 1 significantly correlated with measured sodium excretion using 24 h urine collection among the population (R = 0.54, P < 0.01). They also performed an additional validity examination using external 513 people who did not participate in the INTERSALT study. Consequently, they reported that the difference between measured and estimated values was larger in quintile groups with a smaller amount of sodium excretion. They mentioned that the method was nonetheless considered to be useful to estimate sodium excretion of subjects with relatively high salt intake, because the differences were smaller in the quintile groups with larger amounts of sodium excretion.

In the present data, using Tanaka’s method, estimated sodium excretion significantly correlated with measured sodium excretion (R = 0.52, P < 0.01). Such correlation coefficient was nearly the same as Tanaka’s data. Patients with a low eGFR (stage 4 and 5 CKD) had a relatively stronger correlation (R = 0.60, P < 0.01) than patients with a moderate to high eGFR. Moreover, the assessments of performance for urinary sodium equation demonstrated that median bias and IQR were lower in patients with eGFR < 30 mL/min. Accuracy, which were estimated by RMSE and P30, were higher in patients at lower levels of eGFR. These results indicated Tanaka’s method had higher predictive value especially in low eGFR patients with CKD.

Sodium excretion exhibits a diurnal rhythm. In the general population, sodium excretion during the nighttime has been reported to be 20% lower than during the daytime [16,17]. Moreover, sodium excretion was reported to be higher in the afternoon than in the morning [18]. Several studies have demonstrated that the 24 h Na/Cr ratio was markedly correlated with the spot urine Na/Cr ratio obtained in the late afternoon, not in the morning [19,20]. In the present study, most of the spot urine samples were collected in the morning. The moderate correlation between measured and estimated sodium excretion (R = 0.52) was probably due to the difference of the time to submit spot urine. Fukuda et al. [21] investigated the impact of renal function on the night/day ratio of blood pressure and urinary water and electrolyte excretion. They demonstrated in patients with glomerulopathy that as renal function deteriorated, nocturnal blood pressure elevated and urinary excretion rates of sodium and protein were enhanced during the night. The present study revealed that estimated sodium excretion tended to have a higher accuracy to assess measured (real) salt intake in patients with a low eGFR. Considering these findings, the low eGFR could have muted the diurnal fluctuation of sodium excretion, and stabilized the predicted value.

Finally, the sensitivity and specificity of estimated sodium excretion were analyzed in relationship to measured sodium excretion with two cut-off points (>170 and >100 mEq/day). International recommendations suggest that the salt intake of the general population should be less than 5-6 g (100 mEq)/day [22,23]. On the other hand, while salt intake in Japan is decreasing, it is still high, at about 11 g/day. Kuriyama et al reported reduction in salt intake from 11-14 g/day to 7-8 g/day led to a significant decrease in daily protein excretion in 51 Japanese CKD patients [10]. Swift et al reported that reducing salt intake from 170 mEq/gay to 89 mEq/day reduced blood pressure and urine protein excretion in black hypertensives [24]. Considering these findings, the two cut-off points were set at >170 mEq (10 g) /day and >100 mEq (6 g) /day. Estimated sodium excretion using spot urine had high accuracy to predict measured sodium excretion when the cut-off point was >170 mEq/day (AUC: 0.835). These results agreed with Tanaka’s assessment that the equation was considered to be useful to estimate sodium excretion of subjects with relatively high salt intake.

There were some limitations in the present study. The number of patients recruited was relatively small. Moreover, number of patients with a low eGFR (stage 4 and 5 CKD) was smaller than patients with a moderate or high eGFR (stage 1 to 3 CKD). Further studies with larger numbers of patients allocated equally by eGFR may be needed to clarify these issues.

## Conclusions

The present study demonstrated that salt intake could be estimated by spot urine samples, especially in CKD patients with relatively high salt intake and with a low eGFR. Although measurement of 24 h urine sodium excretion is considered the gold standard, assessment of salt intake in spot urine samples could be applied, in part, for management of patients with CKD.

## Abbreviations

CKD: Chronic Kidney Disease; eGFR: Estimated Glomerular Filtration Rate; Cr: Creatinine; Pr.UCr24: 24 h Urinary Cr Excretion; IQR: Inter quartile range; P_30_: Proportion of estimates within 30% deviation of measured sodium excretion; RMSE: Root mean square error; ROC: Receiver-Operating Characteristic; AUC: Area Under the Curve; CI: Confidence Intervals.

## Competing interests

All the authors declare no competing interests.

## Authors’ contributions

MO analyzed the data in this study, and all authors interpreted the data. All authors contributed substantially to the writing of this manuscript and read and approved the final manuscript.

## Pre-publication history

The pre-publication history for this paper can be accessed here:

http://www.biomedcentral.com/1471-2369/13/36/prepub
